# Pneumococcal genetic variability in age-dependent bacterial carriage

**DOI:** 10.7554/eLife.69244

**Published:** 2022-07-26

**Authors:** Philip HC Kremer, Bart Ferwerda, Hester J Bootsma, Nienke Y Rots, Alienke J Wijmenga-Monsuur, Elisabeth AM Sanders, Krzysztof Trzciński, Anne L Wyllie, Paul Turner, Arie van der Ende, Matthijs C Brouwer, Stephen D Bentley, Diederik van de Beek, John A Lees

**Affiliations:** 1 Department of Neurology, Amsterdam UMC, University of Amsterdam Meibergdreef Netherlands; 2 Department of Clinical Epidemiology, Biostatistics and Bioinformatics, University of Amsterdam Amsterdam Netherlands; 3 Centre for Infectious Disease Control, National Institute for Public Health and the Environment Bilthoven Netherlands; 4 Department of Pediatric Immunology and Infectious D, Wilhelmina Children's Hospital Utrecht Netherlands; 5 Epidemiology of Microbial Diseases, Yale School of Public Health New Haven United States; 6 Cambodia Oxford Medical Research Unit, Angkor Hospital for Children Siem Reap Cambodia; 7 Centre for Tropical Medicine and Global Health, Nuffield Department of Medicine, University of Oxford Oxford United Kingdom; 8 Department of Medical Microbiology and Infection Prevention, Amsterdam UMC Amsterdam Netherlands; 9 The Netherlands Reference Laboratory for Bacterial Meningitis Amsterdam Netherlands; 10 Parasites and Microbes, Wellcome Sanger Institute Cambridge United Kingdom; 11 European Molecular Biology Laboratory–European Bioinformatics Institute Cambridge United Kingdom; 12 MRC Centre for Global Infectious Disease Analysis, Department of Infectious Disease Epidemiology, Imperial College London London United Kingdom; University of the Witwatersrand South Africa; University of the Witwatersrand South Africa

**Keywords:** *Streptococcus pneumoniae*, pneumococcus, meningitis, genetics, microbial, Other

## Abstract

The characteristics of pneumococcal carriage vary between infants and adults. Host immune factors have been shown to contribute to these age-specific differences, but the role of pathogen sequence variation is currently less well-known. Identification of age-associated pathogen genetic factors could leadto improved vaccine formulations. We therefore performed genome sequencing in a large carriage cohort of children and adults and combined this with data from an existing age-stratified carriage study. We compiled a dictionary of pathogen genetic variation, including serotype, strain, sequence elements, single-nucleotide polymorphisms (SNPs), and clusters of orthologous genes (COGs) for each cohort – all of which were used in a genome-wide association with host age. Age-dependent colonization showed weak evidence of being heritable in the first cohort (*h*^2^ = 0.10, 95% CI 0.00–0.69) and stronger evidence in the second cohort (*h*^2^ = 0.56, 95% CI 0.23–0.87). We found that serotypes and genetic background (strain) explained a proportion of the heritability in the first cohort (*h*^2^_serotype_ = 0.07, 95% CI 0.04–0.14 and *h*^2^_GPSC_ = 0.06, 95% CI 0.03–0.13) and the second cohort (*h*^2^_serotype_ = 0.11, 95% CI 0.05–0.21 and *h*^2^_GPSC_ = 0.20, 95% CI 0.12–0.31). In a meta-analysis of these cohorts, we found one candidate association (p=1.2 × 10^-9^) upstream of an accessory Sec-dependent serine-rich glycoprotein adhesin. Overall, while we did find a small effect of pathogen genome variation on pneumococcal carriage between child and adult hosts, this was variable between populations and does not appear to be caused by strong effects of individual genes. This supports proposals for adaptive future vaccination strategies that are primarily targeted at dominant circulating serotypes and tailored to the composition of the pathogen populations.

## Introduction

*Streptococcus pneumoniae* is a common commensal of the human upper respiratory tract and nasopharynx, but can also cause pneumonia and invasive diseases such as sepsis or meningitis (Bogaert et al., 2004). Invasive pneumococcal disease (IPD) has a high mortality, and the overall mortality rate from IPD is higher in extreme age ranges, such as infants and the elderly (Wahl et al., 2018; O Brien et al., 2009). In the Netherlands, pneumococcal carriage rates are higher in children than in adults, with a prevalence of up to 80% at 2 years of age (Wyllie et al., 2016).

Pneumococcal carriage manifests as multiple carriage episodes of different serotypes. From birth, mucosal immunity builds up against different serotypes due to exposure, while immunity from maternal antibodies wanes. Host age is known to affect carriage prevalence and carriage duration of different serotypes (Stearns et al., 2015; Turner et al., 2012b), which is suggested to be driven by differences in immunity.(Wyllie et al., 2020) Studies in mice and humans showed evidence for age-dependent host–pathogen interactions involving interleukin (IL)-1 response in reaction to the pore-forming pneumolysin (*ply*) toxin (Kuipers et al., 2018). IgA secretion is important in clearing *S. pneumoniae* from host upper respiratory tract mucosa, and this secretion is more effective in previously exposed individuals, the adults (Binsker et al., 2020). Bacterial genetics has been shown to explain over 60% of the variability in carriage duration, and specifically that the presence of a bacteriophage inserted in a mediator of genomic competence was associated with a decreased carriage duration (Lees et al., 2017b).

Pneumococci are highly genetically variable, displaying over 100 diverse capsular serotypes (Ganaie et al., 2020), which are a major antigen and the strongest predictor of carriage prevalence (Croucher et al., 2018). Pneumococcal conjugate vaccines (PCVs), targeting up to 13 capsule serotypes with high burden of invasive disease, decrease the rate of nasopharyngeal carriage and invasive disease (Whitney et al., 2003; Poehling et al., 2006). Besides a direct effect of vaccination with a PCV on the disease burden in the target population, that is, young children, it also reduces the disease burden caused by pneumococci with vaccine serotypes in the population not eligible for vaccination through indirect protection from colonization – reducing carriage rates in children reduces overall transmission of the most invasive serotypes (Croucher et al., 2018; Desai et al., 2015; von Gottberg et al., 2014). However, the introduction of PCV has resulted in the replacement of serotypes not covered by the vaccine (Croucher et al., 2013; Corander et al., 2017), which in some countries reaches levels of invasive disease return towards pre-vaccine levels (Ladhani et al., 2018; Koelman et al., 2020).

As not all serotypes can be included in a conjugate vaccine, three perspectives leading to improved pneumococcal vaccination have been proposed: whole-cell vaccines (Malley et al., 2001; Campo et al., 2018), protein vaccines (Moffitt and Malley, 2016), or changing components in the conjugate vaccine in response to the circulating population.(Colijn et al., 2020) Whole-cell vaccination trials are ongoing, but efficacy remains unproven in human populations (Morais et al., 2019). Protein vaccines contain antigens that elicit a strong mucosal immune response, with their targets chosen to be common or conserved in the target population, and ideally reducing onward transmission (Pichichero, 2017). In their current form, protein vaccines are not thought to be effective on their own, but if administered with serotype conjugates (possibly by replacing the carrier protein) they may help to reduce serotype replacement. Detailed modeling of the dynamics of pneumococcal population genetics has shown that targeting these vaccines towards serotypes prevalent in specific populations would likely be a superior strategy. This work further shows that providing age-specific vaccine design using complementary adult-administered vaccines (CAVs) is predicted to have the greatest effect on total IPD burden (Colijn et al., 2020). These authors also modeled including pilus in the vaccine, which is more frequently present in a few key invasive serotypes and in infant carriage, but found this to be inferior to conjugate vaccines.

If proposing a future pneumococcal vaccination strategy based on host age, we should aim to better understand the differences between infant and adult carriage. Differences between other host niches have been found, some with a potential effect on onward transmission (Lees et al., 2017c; Lees et al., 2017a; Zafar et al., 2019). In particular, a previous study has suggested that the presence of pilus, which is found in a minority of pneumococcal isolates, has a selective advantage in infant carriage (Binsker et al., 2020).

We therefore wished to test three hypotheses. Firstly, carriage rates of individual strains or serotypes vary substantially between infants and adults in the same contact networks. Secondly, this variation is attributable at least in part due to pathogen genetic adaptation to either the infant or adult nasopharynx, which are immunologically different niches. Finally, this adaptation is due both to serotype and genetic background, and that some of the genetic effects are resolvable to individual genes, alleles, or regulatory variants that arise on multiple different genetic backgrounds due to a selective advantage. If we find clear associations, this would support proposals for age-specific vaccine design and may also suggest specific protein components that more broadly suppress carriage in the target age group than multivalent PCV alone.

For the first hypothesis, to test varying rates of carriage we can swab children and adults from the same population (and likely exposed to the same infection pressures) and quantify serotype and strain prevalence. For the second hypothesis, if we whole-genome sequence bacteria from these swabs, we can model the effects of all genomic variants on host age in a heritability analysis. This accounts for genetic variation that arose long ago and is fixed in particular lineage, but cannot map it to a particular region of the genome (Earle et al., 2016). To find individual genetic effects, which have necessarily occurred more recently and frequently, one study design would be to identify adult–infant transmission pairs and find variation that consistently occurs in localized regions of the genome, which would be particularly informative if also associated with a particular direction of transmission (Lees et al., 2017c). This removes genetic background as a confounder, giving a clean signal (Young et al., 2012). However, the identification of such pairs is very challenging for *S. pneumoniae*, and even when possible the small numbers limit power. We propose taking the more ‘opportunistic’ approach taken in genome-wide association studies (GWAS), where as many cases and controls (in this case, infant and adult samples) as possible are accumulated to boost statistical power, and genetic background is then controlled for in the association analysis. Where variation associated with age has arisen independently on multiple genetic backgrounds, GWAS has the ability to find these signals among the many lineage associations tested in the second step. In all cases, analysis can be improved by studying more than one population to determine whether findings are consistent among different host and pathogen populations.

To carry out these analyses, we used pneumococci isolated from nasopharyngeal swabs of 4320 infants and adults from the Netherlands (2009–2013) and Myanmar (October 2007–November 2008). Each cohort contains infant and adult samples from carriage, and there are significant differences between the host populations. This allows us to follow the above approach in each population and compare our findings between the populations. We present our findings with respect to each above hypothesis in turn and interpret them through the lens of using population genomics to determine optimal vaccination strategies.

## Results

We first analyzed the observed distribution of serotypes and strains in each of the two cohorts to assess overall trends of differences in carriage between adults and children exposed to similar forces of infection and look at the pathogen population’s genetic heterogeneity between the two cohorts. Although our cohorts were broadly matched in the primary phenotype, age, large differences between the pathogen population are expected due to different geographies, social backgrounds, and only children in one cohort being vaccinated. Nucleotide variation across the entire genome can be used to cluster genetically related isolates into consistently named strains, called global pneumococcal sequence clusters (GPSC) (Gladstone et al., 2019). We used this over older gene-by-gene approaches such as MLST as it has been shown to represent biologically discrete clusters in the population, uses the full resolution available from whole-genome sequencing, and has strong community support (Gladstone et al., 2020). For each sample, we enumerate the serotype (which is targeted by the vaccine) and GPSC membership, and count the number of each serotype observed in adult and child carriage.

### Serotypes and strains are variably carried between age groups and between cohorts

The Dutch cohort was made up of 1329 *S*. *pneumoniae* isolates comprising 41 unique serotypes (Supplementary file 1). Of these isolates, 689 (52%) comprised seven serotypes: 19A (225; 17%), 11A (111; 8%), 6C (97; 7%), 23B (84; 6%), 10A (67; 5%), 16F (54; 4%), and 21 (51; 4%). In this cohort of which the children were vaccinated, a minority of isolates (101; 8%) belonged to one of the vaccine serotypes (Supplementary file 2). A total of 3085 pneumococcal isolates of the Maela (unvaccinated) cohort comprised 64 unique serotype groups (Supplementary file 3). Of these isolates, 1631 (53%) comprised five serotypes: non-typable (511; 17%), 19F (402, 13%), 23F (307, 10%), 6B (236; 8%), and 14 (175; 6%). In the Dutch cohort, there were 59 unique sequence clusters of which the four largest sequence clusters were GPSC 4 (171; 13%), GPSC 3 (156; 12%), GPSC 7 (131; 10%), and GPSC 11 (119; 9%) (Supplementary file 4). There were 127 unique sequence clusters found in the Maela cohort (Supplementary file 5). The four largest sequence clusters were GPSC 1 (352; 13%), GPSC 28 (190; 7%), GPSC 20 (168; 6%), and GPSC 42 (123; 5%). We also looked at a subset of the Maela cohort, which included only the earliest obtained samples from unique individuals (mothers and children). This subset consisted of 762 isolates, including 380 from mother–child pairs. Isolates in this subset had the same serotypes among the most common serotypes as in the full dataset (Supplementary file 6 and Supplementary file 7). Restricting this subset to mother–child paired samples only included the same serotypes and sequence clusters among the most prevalent (Supplementary file 6 and Supplementary file 7).

**Figure 1.**
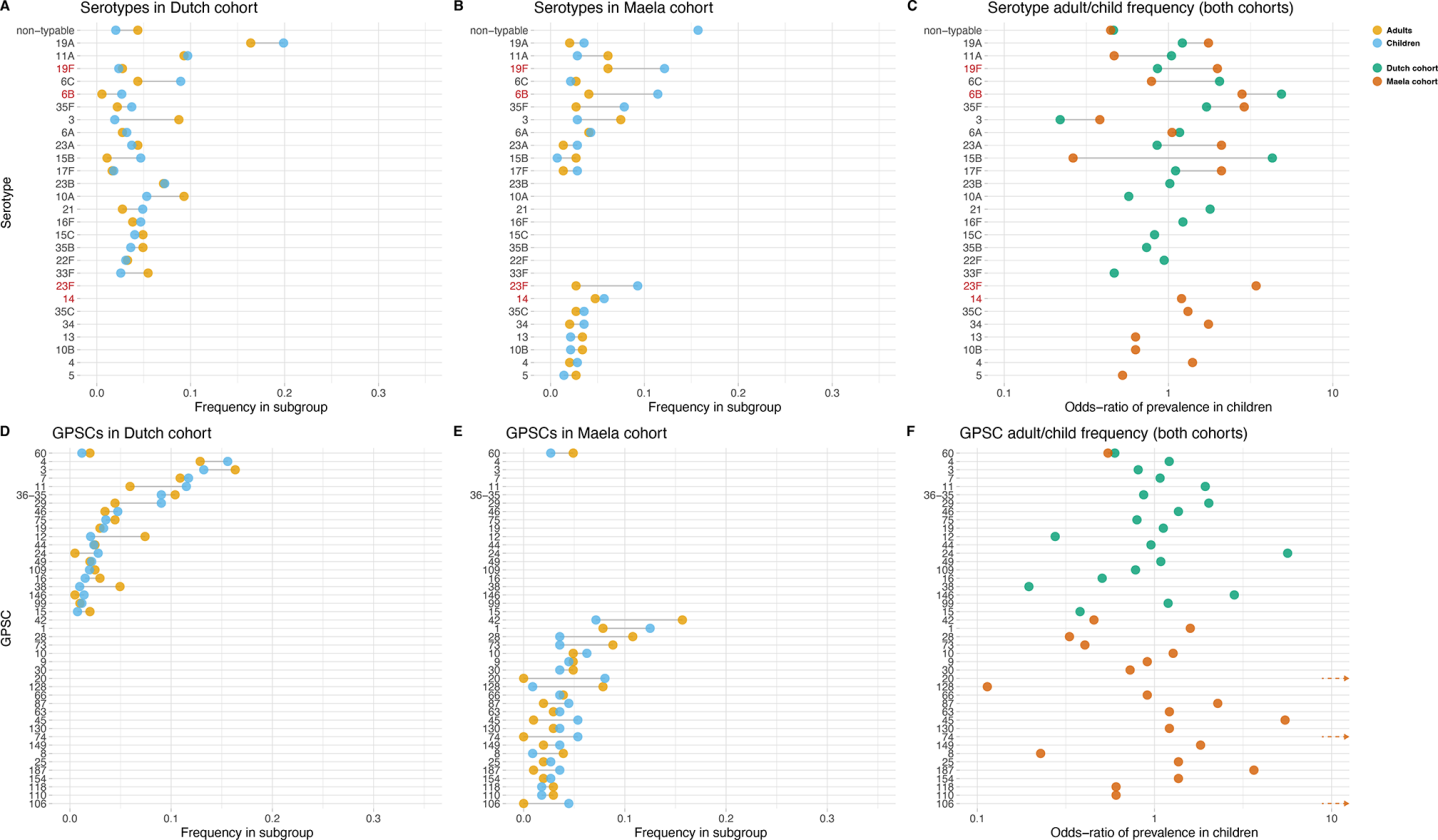
Serotype and strain (global pneumococcal sequence clusters [GPSC]) distribution by age and between cohorts. Blue dots represent frequency of serotype and strain in child carriage, yellow dots represent frequency in adult carriage. Red and green dots show odds ratio of prevalence in children in the Maela and Dutch cohorts, respectively, on a log scale for serotype. Lines show differences. Top row: dominant serotypes, ordered by presence in cohort, and internally by overall frequency. Vaccine serotypes shown in red. (**A**) Serotype frequency in the Dutch cohort. (**B**) Serotype frequency in the Maela cohort. (**C**) Comparison of adult/child log odds in each cohort for serotype. Second row: dominants strains (GPSCs), ordered by presence in cohort, and internally by overall frequency. (**D**) Strain frequency in Dutch cohort. (**E**) Strain frequency in Maela cohort. (**F**) Comparison of adult/child log odds in each cohort for strain.

Some serotypes exhibited a large difference in colonization frequency between the two age groups (Figure 1). In the Dutch cohort, serotype 6C (chi-squared test, p=0.02, not corrected for multiple testing) and serotype 15B (p=0.02) were overrepresented in children relative to adults, serotype 3 was overrepresented in adults relative to children (p=2.5 × 10^–5^), while in the Maela cohort, serotype groups overrepresented in children were serotype 23F (chi-squared test, p=0.04) and serotype 6B (chi-squared test, p=0.04); while non-typeable serogroup was overrepresented in adults (chi-squared test, p=3.0 × 10^–4^) (Table 1). None of the 20 largest groups of sequence clusters overlapped between the cohorts. In the Dutch cohort, only GPSC 11 was significantly associated with carriage in children (chi-squared test, p=0.03, not corrected for multiple testing), while GPSC 12 (chi-squared test, p=1.2 × 10^–4^) and GPSC 38 (chi-squared test, p=2.1 × 10^–4^) were overrepresented in adults. In the Maela cohort, only sequence cluster GPSC 128 was overrepresented in children compared to adults (chi-squared test, p=0.04), while GPSC 20 (chi-squared test, p=7.0 × 10^–3^) and GPSC 74 (chi-squared test, p=0.04) were overrepresented in adults (Table 2).

**Figure 1—figure supplement 1.**
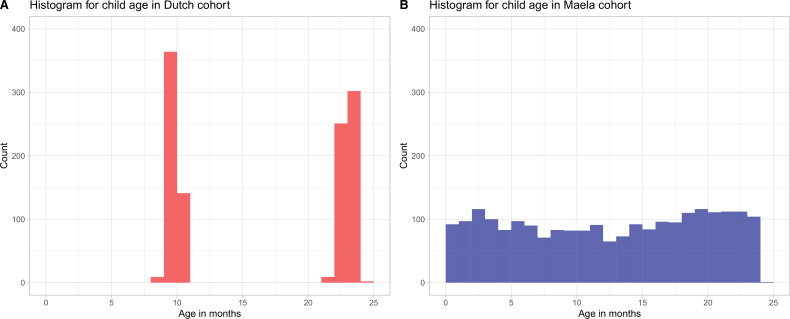
Histogram for child age (in months) in (**A**) Dutch cohort (red bars) and (**B**) Maela cohort (blue bars). Due to the differences in sampling strategies in the studies from which the samples were obtained, children in the Dutch cohort were sampled at ages 9–11 months and 22–24 months, while in the Maela cohort children were sampled in the age range 1–24 months.

**Table 1.** Chi-squared values for serotypes in the Dutch and Maela cohorts and the age group that the serotype is affiliated with.

Serotype	Dutch cohort		Maela cohort	
	χ^2^ p-value	Age group	χ^2^ p-value	Age group
Non-typeable	0.188	Adults	3.0 × 10^–4^	Adults
19A	0.089	Children	0.690	Children
11A	0.591	Children	0285	Adults
19F	1	Adults	0.131	Children
6C	0.022	Children	1	Adults
6B	0.099	Children	0.040	Children
35F	0.279	Children	0.100	Children
3	2.5 × 10^–5^	Adults	0.129	Adults
6A	0.709	Children	1	Children
23A	1	Adults	-	-
15B	0.023	Children	-	-
17F	0.943	Children	-	-
23B	0.727	Children	-	-
10A	0.155	Adults	-	-
15C	1.000	Adults	-	-
35B	0.775	Adults	-	-
22F	1	Adults	-	-
33F	0.132	Adults	-	-
23F	-	-	0.040	Children
14	-	-	0.949	Children
35C	-	-	0.961	Children
34	-	-	0.690	Children
13	-	-	0.756	Adults
10B	-	-	0.756	Adults
4	-	-	0.966	Children
5	-	-	0.710	Adults
33B	-	-	1	Children
28F	-	-	0.652	Children
19B	-	-	0.710	Adults
7F	-	-	0.971	Children
20	-	-	0.971	Children
18C	-	-	1	Adults

χ^2^, chi-square; -, not applicable.

**Table 2.** Chi-squared values for strains in the Dutch and Maela cohorts and the age group that the strain is affiliated with.

GPSC	Dutch cohort		Maela cohort	
	χ^2^ p-value	Age group	χ^2^ p-value	Age group
60	0.568	Adults	0.727	Adults
4	0.298	Children	-	-
3	0.392	Adults	-	-
7	0.858	Children	-	-
11	0.03	Children	-	-
35 and 36	0.617	Adults	-	-
29	0.049	Children	-	-
46	0.563	Children	-	-
75	0.666	Adults	-	-
19	0.978	Children	-	-
12	1.2 × 10^–4^	Adults	-	-
44	1	Adults	-	-
24	0.094	Children	-	-
49	1	Children	-	-
109	0.817	Adults	-	-
16	0.249	Adults	-	-
38	2.1 × 10^–4^	Adults	-	-
146	0.489	Children	-	-
99	1	Children	-	-
15	0.22	Adults	-	-
42	-	-	0.134	Children
1	-	-	0.276	Adults
28	-	-	0.110	Children
73	-	-	0.253	Children
10	-	-	0.777	Adults
9	-	-	1	Children
30	-	-	0.993	Children
20	-	-	7.0 × 10^–3^	Adults
128	-	-	0.042	Children
66	-	-	1	Children
87	-	-	0.450	Adults
63	-	-	1	Adults
45	-	-	0.129	Adults
130	-	-	1	Adults
74	-	-	0.040	Adults
149	-	-	0.686	Adults
8	-	-	0.364	Children
25	-	-	1	Adults
187	-	-	0.371	Adults
154	-	-	1	Adults
118	-	-	0995	Children
110	-	-	0.995	Children
106	-	-	0.073	Adults

χ^2^, chi-square; -, not applicable; GPSC, global pneumococcal sequence clusters.

**Figure 2.**
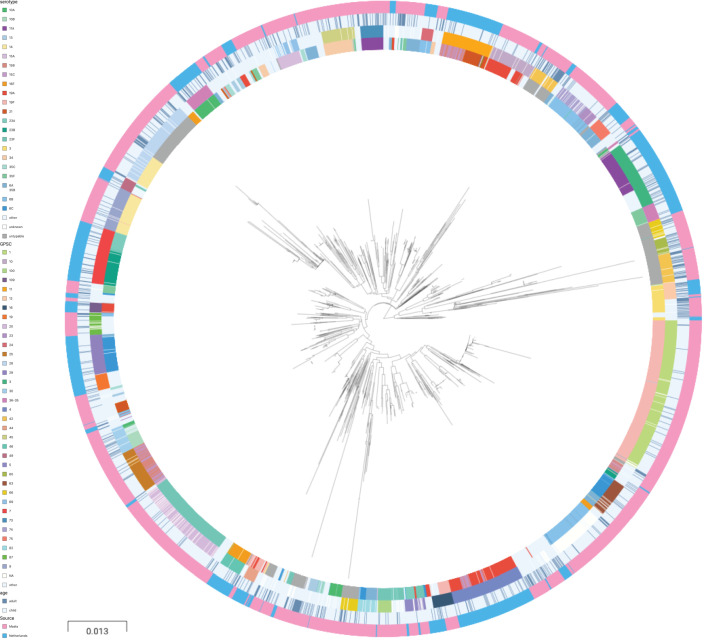
Phylogenetic tree of carriage samples from both cohorts. The rings show metadata for the samples. Depicted from inside to outside, these are serotype, sequence cluster (global pneumococcal sequence clusters [GPSC]), age, and source (Maela, Netherlands). Scale bar: 0.013 substitutions per site. An interactive version is available at here (project link available here).

A phylogenetic tree of pooled sequences from both cohorts, with serotype, sequence cluster, age group, and cohort for each sequence, revealed clonal discrimination between cohorts (Figure 2). Combined with the effects shown in Figure 1, this highlighted a key feature of our analysis of these datasets, which was the genetic heterogeneity between the two cohorts. Individually, each dataset clearly has strains and serotypes with strong signals of host age differences, but the overall makeup of each dataset is very different (nine common serotypes are shared, but only a single common GPSC), and where there are shared serotypes these can have different effect directions between the two cohorts.

**Figure 2—figure supplement 1.**
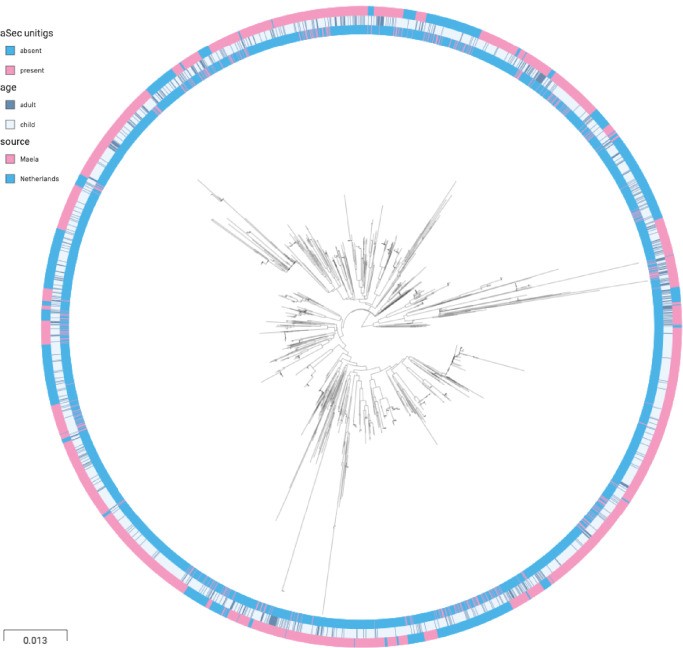
Phylogenetic tree of carriage samples from both cohorts. The rings show metadata for the samples. Depicted from inside to outside, these are presence or absence of the unitig upstream to the aSec gene, age (adult or child), and source (Maela or Netherlands). Scale bar: 0.013 substitutions per site.

### Host age is heritable and mostly explained by strain and serotype

**Figure 3.**
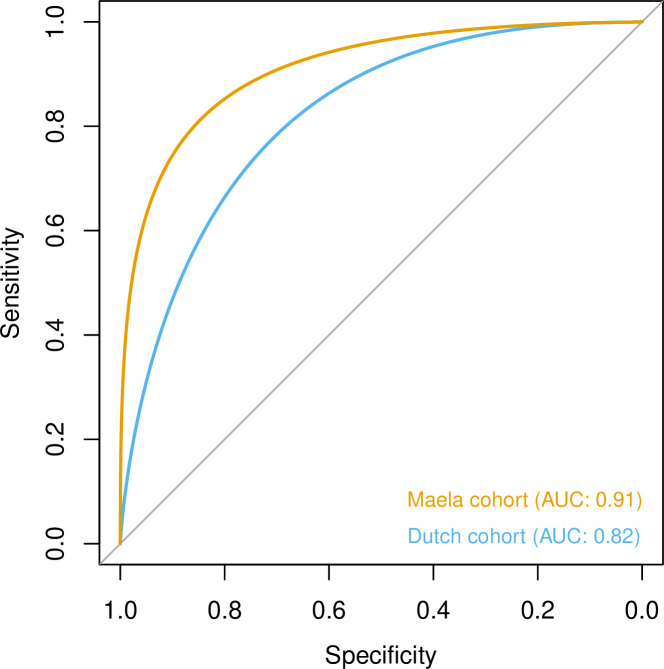
Prediction of host age from pan-genomic variation in each cohort. The smoothed receiver-operating characteristic (ROC) curve based on a linear predictor (elastic net fitted to unitigs, with strains used as folds for cross-validation) is shown. Area under the curve (AUC) is 0.5 for no predictive ability and 1 for perfect prediction.

To quantify the amount of variability in carriage age explained by variability in the genome, we calculated a heritability estimate (*h*^2^) for each cohort. For isolates in the Dutch cohort, we did not find strong evidence that genetic variability in bacteria was related to variance in host age (*h*^2^ = 0.10, 95% CI 0.00–0.69). In the Maela cohort, we found significant evidence that affinity with host age was heritable (*h*^2^ = 0.56, 95% CI 0.23–0.87) and thus genetic variation in this cohort explained variation in carriage age to a greater degree. In both cohorts, pan-genomic variation could be used to predict host age to some degree of accuracy (area under the receiver-operating characteristic [ROC] curve 0.82 [Dutch cohort]; 0.91 [Maela cohort]), suggestive of some level of heritability and association of host age with strain (Figure 3). Prediction between cohorts using a simple linear model failed as the genetic variants chosen as predictors were not found in the other cohort – again highlighting the high level of genetic heterogeneity between cohorts.

To further investigate the association of serotype and sequence cluster to carriage age, we determined the proportion of variation in carriage age explained by serotype and sequence cluster alone. Here, we estimated *h*^2^_serotype_ = 0.07 (95% CI 0.04–0.14) and *h*^2^_GPSC_ = 0.06 (95% CI 0.03–0.13) for the Dutch cohort and *h*^2^_serotype_ = 0.11 (95% CI 0.05–0.21) and *h*^2^_GPSC_ = 0.20 (95% CI 0.12–0.31) for the Maela cohort, confirming a larger contribution of serotype and sequence cluster to carriage age heritability in sequences from the Dutch cohort. We also performed a genome-wide association analysis, but without controlling for population structure. This reveals genetic variants specific to serotype as determinants for carriage age (p-values<5.0 × 10^–8^) in both cohorts (Supplementary file 8, Dutch cohort, and Supplementary file 9, Maela cohort). Among the genetic variants with the lowest p-values were variants in capsule locus genes (Cps) in both cohorts. This further supports a role of strain and serotype in association with host age, but does not distinguish between the two.

### Genome-wide association analysis does not find genetic variants independent of strain

**Figure 4.**
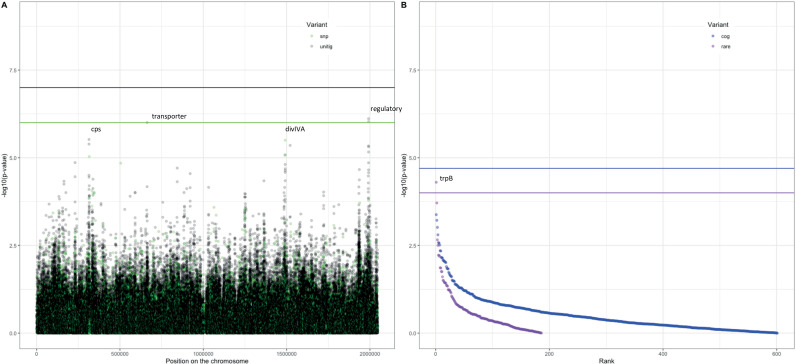
Association of variants after meta-analysis with carriage age 0–24 months. (**A**) Minus log-transformed p-value on the y-axis and position of unitig and single-nucleotide polymorphism (SNP) variants on the *S. pneumoniae* genome on the x-axis (Manhattan plot). (**B**) Minus log-transformed p-value on the y-axis and sorted lowest to highest p-value for rare variant burden in genes (purple) and clusters of orthologous genes (COGs, blue) on the x-axis.

Following these observations that serotype and strain do not explain the full heritability, specifically in the Maela cohort, we performed a pathogen genome-wide association analysis to investigate whether we can detect genetic variants irrespective of the genetic background that are associated with carriage in children or adults. Though the cohorts have little genetic overlap in terms of genetic background, we would be well-powered to detect genetic variation independent of background (‘locus’ associations) (Earle et al., 2016; Lees et al., 2016). In the Dutch cohort, none of the unitigs, SNPs, COGs, or rare variants surpassed the threshold for multiple testing correction (Figure 4—figure supplement 1). The burden (sum) of rare variants in a gene for tryptophan synthase, *trpB*, approaches the multiple testing threshold, but was not significant. In the Maela cohort, unitigs in the *ugpA* gene surpassed the threshold for statistical significance (Figure 4—figure supplement 2), but these did not hold after meta-analysis. After meta-analysis, there were two hits that surpassed the threshold for statistical significance (Figure 4).

**Figure 4—figure supplement 1.**
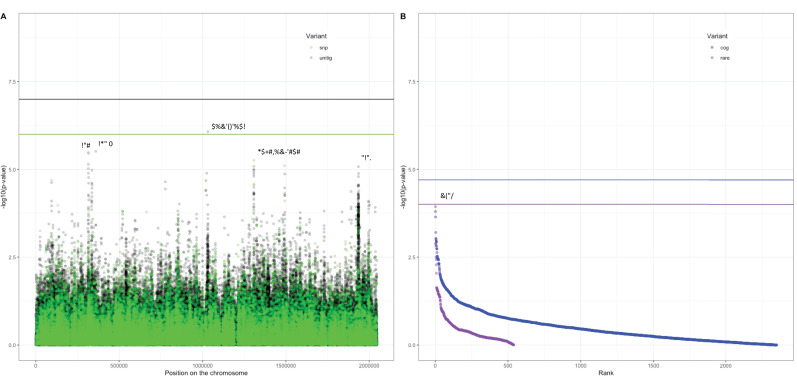
Association of variants in the Dutch cohort with carriage age 0–24 months. (**A**) Minus log-transformed p-value on the y-axis and position of unitig and single-nucleotide polymorphism (SNP) variants on the *S. pneumoniae* genome on the x-axis (Manhattan plot). (**B**) Minus log-transformed p-value on the y-axis and sorted lowest to highest p-value for rare variant burden in genes (purple) and clusters of orthologous genes (COGs, blue) on the x-axis. Variants of interest have annotations added.

**Figure 4—figure supplement 2.**
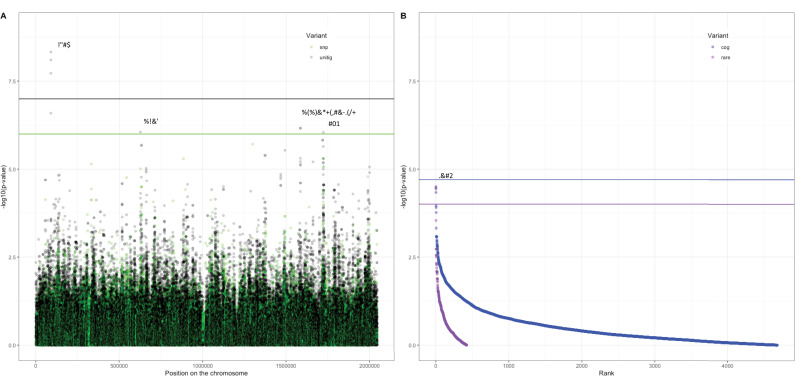
Association of variants in the Maela cohort with carriage age 0–24 months. (**A**) Minus log-transformed p-value on the y-axis and position of unitig and single-nucleotide polymorphism (SNP) variants on the *S. pneumoniae* genome on the x-axis (Manhattan plot). (**B**) Minus log-transformed p-value on the y-axis and sorted lowest to highest p-value for rare variant burden in genes (purple) and clusters of orthologous genes (COGs, blue) on the x-axis. Variants of interest have annotations added.

The first is a nucleotide sequence marked by multiple unitigs of which the lowest has a p-value of 1.2 × 10^–9^ (Supplementary file 10). This sequence does not map to the *S. pneumoniae* D39V reference sequence (Altschul et al., 1990) For this reason, it is not visualized on the Manhattan plot, for which unitigs were mapped to the *S. pneumoniae* D39V reference sequence (Figure 4). Upon inspection of the individual sequences these unitigs are called from, we find them to map in the intergenic region between open-reading frames encoding the accessory Sec-dependent serine-rich glycoprotein adhesin and a MarR-like regulator, respectively. This region contains sequences resembling transposable elements and an open-reading frame encoding a transposase. The unitigs map upstream of the start codon of the accessory Sec-dependent serine-rich glycoprotein adhesin. The sequence is present in 169 out of 1282 (13%) sequences in the Dutch cohort and in 241 out of 3085 (8%) in the Maela cohort. The sequence is present in isolates dispersed over the phylogenetic tree and associated with carriage in children (Figure 2—figure supplement 1). This protein is involved in adhesion to epithelial cells and biofilm formation (Chan et al., 2020; Middleton et al., 2021; Weiser et al., 2018). Given that this sequence lies just upstream of the start codon, it is plausible that variation of this sequence alters the expression of the Sec-dependent adhesin protein, and therefore affects carriage.

The second hit is a burden of rare variants in a gene for tryptophan synthase, *trpB*, that surpass the threshold for statistical significance at a p-value of 5.0 × 10^–5^. The variants are two frameshift variants of very low frequency. These result in a predicted dysfunctional *trpB* gene in 9 out of 1282 (1%) sequences in the Dutch cohort and in 12 out of 3073 (0.4%) sequences in the Maela cohort. This association of the *trpB* gene is likely to be an artifact of low allele frequency as we estimate we are only powered to detect variation in at least 5% of isolates.

### Pilus gene presence does not determine carriage age independent of genetic background

Finally, we investigated whether pneumococcal isolates containing a pilus gene preferentially colonize children in the Dutch cohort, as has been previously described in the Maela cohort (Binsker et al., 2020; Turner et al., 2012a). This study analyzed the Maela cohort and found that 934 out of 2557 (37%) isolates in children versus 95 out of 592 (16%) isolates in adults had pilus genes present. However, this association of pilus gene presence to carriage age was dependent on lineages within the population (Binsker et al., 2020). In the Dutch cohort, we found no evidence that host age was dependent on pilus gene presence (22 out of 208 [10%] in adults versus 129 out of 1099 [12%] in children). This was the case whether or not the genetic background was adjusted for (p=0.35, uncorrected for population structure, and p=0.69, corrected for population structure). Based on these findings, we suggest that the previously reported pilus-IgA1 association is not a universal explanation for difference in colonization between hosts of different ages.

## Discussion

The age of the host is known to have an important effect on pneumococcal colonization (Croucher et al., 2018). Observational studies have demonstrated variation in serotype prevalence and carriage duration between infants and adults. Mechanistic studies in mice and humans have shown examples of differing immune responses depending both on host factors and pathogen factors. Findings from these studies include the observation that capsular polysaccharides (determinants of serotype) inhibit phagocytic clearance in animal models of upper respiratory tract colonization (Nelson et al., 2007). A pneumolysin-induced IL-1 response determined colonization persistence in an age-dependent manner Kuipers et al., 2018; and pilus-expressing strains were found to preferentially colonize children because of immune exclusion via secretory IgA in non-naïve hosts (Binsker et al., 2020).

Building upon these observations, we sought to investigate and quantify the contribution of pathogen genetic variation to carriage in infant versus adult hosts using a top-down approach to systematically test the effect of pathogen genome variation on niche specificity (with age as the niche). We used whole-genome sequencing and applied statistical genetic methods to two large *S. pneumoniae* carriage cohorts. We aimed to quantify the differing patterns of serotype and strain prevalence between the two age classes during carriage and search for other genetic factors associated with host age. We show evidence that bacterial genetic variability indeed influences predilection for host age, though the effect size appears to be highly variable between populations.

Strain, or genetic background, explains 60% of the total heritability in the Dutch cohort, but only a minority in the Maela cohort. We found sequences in one region that map closely to the start codon of the accessory Sec-dependent serine-rich glycoprotein adhesin to be associated with carriage age independent of genetic background, in a meta-analysis of the two cohorts.

In previous bacterial GWAS of antimicrobial resistance (such as a single gene that causes antibiotic resistance), large monogenic effects have typically been found to have high heritabilities close to one, and the GWAS identify the causal variant precisely.(Earle et al., 2016; Chewapreecha et al., 2014b; Lees et al., 2020). When applied to virulence and carriage duration phenotypes, heritable effects have also been found, but these only explained some of the variation in the phenotype. These appeared to be caused by many weaker effects, known as a polygenic trait, not all of which could be detected using the relatively small cohorts available (Lees et al., 2017b; Lees et al., 2019a). Polygenic effects were also seen in another study on the contribution of genetic variation to disease severity of IPD (Cremers et al., 2019). We found similar results for host age heritability in our two cohorts.

We could not distinguish between genetic background or serotype being the primary effect due to their correlation. We did note a difference in effect size of serotype between the two cohorts, which may make it unlikely to be the single largest effect on host age. This difference in cohorts could be explained by strain/GPSC being the main and consistent effect on host age. As strains are different between cohorts and each serotype appears in multiple strains, combining them in different amounts would create different directions of effect for serotype.

Our results are therefore suggestive of the following genetic architecture for association with host age. Primarily, whether a particular infant is colonized with a pneumococcus, when compared to an adult from the same population, is not due to the pathogen’s genetics. This may be due to technical factors such as detectability related to pathogen load (which varies between adults and children, as well as between serotypes), different local forces of exposure, or other environmental or host factors such as diet (which may affect survival to *trpB*-defective pneumococci). However, some of the variation of these patterns can be explained by the pathogen’s genetics. This appears largely to be driven by the fact that some strains and serotypes are more likely to be found in an infant or adult nasopharynx. In addition, there are likely to be many variants that each contribute a small amount to host age preference, but no single universally important gene or variant (a polygenic trait).

From this study, we cannot say specifically which regions of the genome contain these small effects, but it is useful to rule out recently adaptive variants with individually strong effects. We did not replicate the association of piliated genomes in infant hosts in our newly sequenced cohort, further demonstrating important differences between populations. An important corollary of our work is on future pneumococcal vaccine optimization efforts. A promising approach for future vaccination strategies is to target the different age groups (Colijn et al., 2020). Whether these should consist of the dominant disease-causing serotypes overrepresented in carriage by each age group or whether there are age-specific pathogen proteins that should be included is an open question. Our study therefore suggests that targeting these age groups using serotype makeup alone would be sufficient and supports previous observational and modeling studies that advise targeting the serotype makeup in the vaccine at specific populations to maximize their effect.

Three reasons can contribute to not finding individual effects: a high proportion of the heritability being caused by lineage effects; rare locus effects that could not be detected with the current sample size; and by sampling from a cohort with vaccinated children and unvaccinated adults and comparing with a cohort of unvaccinated children and adults, we had lower power due to the reduced overlap within and between cohorts in pan-genome content. Although differences in vaccination status between cohorts is one plausible explanation for interpreting our findings, we were unable to rule out other factors, for example, a population-specific host effect, geographical differences (Li et al., 2019), or the broad effects of different socioeconomic status between these cohorts. Pneumococcal factors such as differences in detectability due to carriage density could also influence results.

One important difference between our study cohorts was that children from the Dutch cohort were vaccinated, while children from the Maela cohort were not. While our findings demonstrate that vaccinated versus unvaccinated children were colonized with different bacterial serotypes and different sequence clusters, we observed differences in prevalence beyond just the serotypes included in the vaccine. Another difference between the cohorts was that adults from the Dutch cohort were males and females, while adults from the Maela cohort were females only.

In summary, we found an effect of pneumococcal genetics on carriage in children versus adult hosts, which varies between cohorts, and is likely primarily driven by serotype or strain (lineage) effects rather than large population-wide effects of individual genes.

## Materials and methods

### Cohort collection

Cohorts were selected based on availability. The Dutch cohort consists of parent–child paired isolates of carriage samples from individuals obtained from three prospective carriage surveillance studies (Spijkerman et al., 2012; Bosch et al., 2016; van Beek et al., 2017). In these studies, carriage was assessed by conventional culture of nasopharyngeal or oropharyngeal swabs of vaccinated children (11 and 24 months of age) and their parents in 2009, 2010/2011, 2012, and 2013 (Spijkerman et al., 2012). All children were vaccinated with PCV-7 or PHiD-CV10 according to the Dutch national immunization program at 2, 3, 4, and 11 months of age. Vaccination status of the parents was unknown. Exclusion criteria are described elsewhere (Spijkerman et al., 2012; Bosch et al., 2016). Nasopharyngeal swabs were collected from all individuals and oropharyngeal swabs were collected from all adult subjects by trained study personnel using flexible, sterile swabs according to the standard procedures described by the World Health Organization (O Brien et al., 2003). After sampling, swabs were immediately placed in liquid Amies transport medium and transported to the microbiology laboratory at room temperature and cultured within 12 hr. Pneumococcal isolates were identified using conventional methods, as described previously (Trzciński et al., 2013). The Maela cohort consists of samples from people from a camp for displaced persons on the Thailand–Myanmar border, where monthly nasopharyngeal sampling was performed in unvaccinated children (0–24 months old) and their mothers. This cohort consists of mother–child paired samples, some of which were sampled from the same mother or child over multiple time points, and unpaired samples from mothers and children. A subselection from this cohort was made to reflect the first sampled isolates for each mother–child pair and unpaired samples to obtain isolates belonging to unique individuals. Procedures for collecting samples and generating whole-genome sequences have been previously described (Turner et al., 2012b; Chewapreecha et al., 2014b).

### Informed consent

Written informed consent was obtained from both parents of each child participant and from all adult participants. Approval for the 2009 and 2012/2013 studies in children and their parents (NL24116 and NL40288/NTR3613) was received from the National Ethics Committee in the Netherlands (CCMO and METC Noord-Holland). For the 2010/2011 study, a National Ethics Committee in The Netherlands (STEG-METC, Almere) waived the requirement for EC approval. Informed consent for the Maela cohort is described elsewhere (Turner et al., 2012b). Studies were conducted in accordance with the European Statements for Good Clinical Practice and the Declaration of Helsinki of the World Medical Association.

### Host age distribution in sequenced carriage cohorts

In the Dutch cohort, children had a median age of 23 months (interquartile range [IQR] 10–24 months). Adults had a median age of 35 (IQR 32–38) years. In the Maela cohort, the median age of children was 13 months (IQR 6–19 months), and for mothers (women of childbearing age) the exact age was unknown (Figure 1—figure supplement 1; Turner et al., 2012b; Turner et al., 2013) In the Dutch cohort, all children were vaccinated with PCV-7 or PHiD-CV10. None of the members of the Maela cohort had received PCV.

### DNA extraction and whole-genome sequencing

For the Dutch cohort, DNA extraction was performed with the Gentra Puregene Isolation Kit (QIAGEN), and quality control procedures were performed to determine yield and purity. Sequencing was performed using multiplexed libraries on the Illumina HiSeq platform to produce paired-end reads of 100 nucleotides in length (Illumina, San Diego, CA). Quality control involved analysis of contamination with Kraken (version 1.1.1)(Page et al., 2016), number and length of contigs, GC content, and N50 parameter. Sequences for which one or more of these quality control parameters deviated by more than 3 standard deviations from the mean were excluded. Sequences were assembled using a standard assembly pipeline (Page et al., 2016). Assembly statistics can be found in Supplementary file 11. Genome sequences were annotated with PROKKA, version 1.11 (Seemann, 2014). For the Maela cohort, DNA extraction, quality control, and whole-genome sequencing have been described elsewhere (Chewapreecha et al., 2014a). Serotypes were determined from the whole-genome sequence by in-house scripts (Croucher et al., 2011). Sequence clusters (strains) were defined as GPSC using PopPUNK (version 2.2.0) using a previously published reference database (Gladstone et al., 2019; Lees et al., 2019b). For 114 and 401 sequences in the Dutch and Maela cohorts, respectively, the GPSC could not be inferred due to low sequence quality.

### Sequencing characteristics and quality control

A total of 1361 bacterial isolates were sequenced as part of the Dutch cohort. During quality control, 32 sequences were excluded. Of these, 8 belonged to a different pathogen species, 9 had contamination, 14 were excluded based on the number of contigs or genome length, and 1 sequence failed annotation. For 47 sequences, host age was missing. The average length of the sequences was 2,105,305 nucleotides, with a standard deviation of 51,679 nucleotides. The mean number of contigs was 67, range 23–226. The association analyses were performed on 1282 sequences in the Dutch cohort. Of these, 1052 were isolated from children and 230 from adults. There were 3085 sequences available from the Maela cohort. Quality control for this cohort was described previously (Chewapreecha et al., 2014a). There were 2503 sequences isolated from children and 582 from adults. In a subset from the Maela cohort, there were 762 isolates from unique hosts, of which 380 were paired isolates (190 from children and 190 from their mothers). For the determination of the frequency and odds ratio of serotype and GPSCs in children and adults, only the first isolate from each carriage episode for each child was included in the analysis. This resulted in 964 serotypes and 799 GPSCs (165 missing) in children, and 582 serotypes and 508 GPSCs (74 missing) in adults. For adults, chi-squared tests to calculate the p-value for association between serotype and strain with age were performed in R (version 4.0.0).

### Phylogenetic tree

A core genome for sequences from both cohorts together was generated with Roary (version 3.5.0, default parameters) using a 95% sequence identity threshold (Page et al., 2015). A maximum likelihood phylogeny of SNPs in the core genome of all sequenced isolates from both cohorts together was produced with IQ-TREE (version 1.6.5, including fast stochastic tree search algorithm, GTR+I+ G) assuming a general time-reversible model of nucleotide substitution with a discrete γ-distributed rate heterogeneity and the allowance of invariable sites (Nguyen et al., 2015).

### Heritability analysis

Based on the kinship matrix and phenotypes, a heritability estimate was performed in limix (version 3.0.4 with default parameters) for both cohorts separately (Lippert et al., 2014). A confidence interval around the heritability estimate was determined with Accurate LMM-based heritability Bootstrap confidence Intervals (ALBI) based on the eigenvalue decomposed distances in the kinship matrix and the heritability estimate with the gglim package (version 0.0.1) in R (version 4.0.0) (Schweiger et al., 2018). To estimate the proportion of heritability attributable to serotype or strain alone, we calculated the heritability with limix, based on a kinship matrix treating serotypes or strains as genetic variants (Lees et al., 2017b; Lees et al., 2018). Again, a confidence interval around the heritability estimate was determined with ALBI (Schweiger et al., 2018). The code used to perform these analyses is available at https://github.com/philipkremer123/carriage_pneumo_heritability (Kremer, 2022a copy archived at swh:1:rev:73c4fa5c8d24d76945308b2616fbb5572d0c39b4) and https://github.com/johnlees/carriage-age-plots, (Kremer, 2022b copy archived at swh:1:rev:f9477d6b8382fee6926be7fd29b99afc15873fe8).

### Determining bacterial genetic variation: Unitigs, SNPs, and COGs

Using the whole-genome sequence reads from both cohorts, we called SNPs, small insertions and deletions, and SNPs clustered as rare variants (deleterious variants at an allele frequency < 0.01) based on the *S. pneumoniae* D39V reference (CP027540) sequence using the Snippy pipeline (version 4.4.0, default parameters). We determined nonredundant sequence elements (unitigs) from assembled sequences in the Dutch cohort by counting nodes on compacted De Bruijn graphs with Unitig-counter (version 1.0.5, default minimum k-mer length of 31) (Jaillard et al., 2018). These unitigs were called in an indexed set of sequences from the Maela cohort with Unitig-caller (version 1.0.0, default parameters) (Lees et al., 2020). This gave us the distribution of sequences from both cohorts with consistent k-mer definitions, making it possible to run predictive models across cohorts. The same Roary run as was used to generate the core-genome alignment was used to extract accessory COGs (Page et al., 2015).

There were 9,966,794 unitigs counted from combined sequences in the Dutch cohort. Of these, 303,901 passed a minor allele frequency (MAF – the frequency of isolates a genetic sequence, or allele, is identified in) of 0.05 filter and had association testing performed. The 9,966,794 unitigs from the Dutch cohort were called in sequences from the Maela cohort to obtain 726,040 unitigs. Association testing in this group was done for 323,112 unitigs that were present at MAF 0.05 or more. Meta-analysis was performed on 251,733 overlapping unitigs. There were 313,143 SNPs called from sequences in the Dutch cohort, of which 43,556 passed MAF filtering. For the Maela cohort, 382,230 SNPs were called and 53,553 passed the MAF filter. For meta-analysis, 20,118 SNPs had overlapping positions and were included. There were 1997 rare variants called in the Dutch cohort, which were burdened in 538 genes. For the Maela cohort, these numbers were 1997 and 423. Together, 186 genes were included in the meta-analysis. Lastly, 2348 COGs were analyzed in the Dutch cohort and 4678 in the Maela cohort. In the meta-analysis, there were 627 overlapping COGs.

### Genome-wide association study

The association analysis for SNPs, unitigs, rare variants, and COGs was run as a linear mixed model in Pyseer (version 1.1.1), with a minimum MAF of 0.05 (Lees et al., 2018). To correct for population structure, the model included a kinship matrix as covariates, which was calculated from the midpoint rooted phylogenetic tree. An association analysis not corrected for population structure was run with unitigs as sequence elements using a simple fixed-effects model in Pyseer. Rare variants were clustered in their corresponding gene and analyzed in a burden test. Meta-analysis was performed on summary statistics from the Pyseer results files with METAL (version released on August 28, 2018, default parameters) for each variant (Willer et al., 2010). A threshold for association of the phenotype with meta-analyzed variants was determined using a Bonferroni correction with alpha < 0.05 and the number of independent tests in the Dutch cohort, giving p<1.0 × 10^–7^ for unitigs, p<1.0 × 10^–6^ for SNPs, p<2.0 × 10^–5^ for COGs, and p<1.0 × 10^–4^ for rare variants. Unitigs were mapped to the *S. pneumoniae* D39V reference genome with bowtie-2 (version 2.2.3, with equal quality values and length of seed substrings 7 nucleotides). In accordance with the study populations in both cohorts, the phenotype was dichotomized as host age 0–24 months versus adult age. Manhattan plots were generated in R (version 3.5.1) with the package ggplot2 (version 3.1.0). Presence or absence of pilus genes was detected by nucleotide BLAST (version 2.6.0, default parameters) analysis. Pilus gene presence association to carriage age was calculated with a likelihood ratio test in Pyseer (version 1.1.1), corrected for population structure by including a kinship matrix as covariates.

The prediction analysis used the elastic net mode of Pyseer. This fitted an elastic net model with a default mixing parameter (0.0069 L1/L2) to the unitigs counted in each cohort using the strains from PopPUNK as folds to try and reduce overfitting (Lees et al., 2020). ROC curves for each cohort were drawn using the linear link output, with the R package pROC (version 1.16.2) using smoothing. To test inter-cohort prediction, the called unitigs from the other cohorts were used as predictors with the model from the opposing cohort.

## Data Availability

Fastq sequences of bacterial isolates from the Dutch cohort were deposited in the European Nucleotide Archive (ENA, study and accession numbers in Supplementary file 12). Sequences of bacterial isolates in the Maela cohort are available at ENA under study numbers ERP000435, ERP000483, ERP000485, ERP000487, ERP000598 and ERP000599 (Supplementary file 13). Summary statistics for the results from the genome wide association studies can be found at https://figshare.com/articles/dataset/S_pneumoniae_carriage_GWAS/14431313. The following previously published dataset was used: KremerPHC 2020*Streptococcus pneumoniae* evolution and population structure during longitudinal sampling in a defined human populationENAPRJEB2357

## Additional files

Supplementary file 1. Serotypes in the Dutch cohort, and the number of samples isolated from child or adult.

Supplementary file 2. Number of samples for each of the vaccine serotypes found in the Dutch cohort.

Supplementary file 3. Serotypes in the Maela cohort, and the number of samples isolated from child or mother.

Supplementary file 4. Strains (global pneumococcal sequence clusters [GPSCs]) in the Dutch cohort, and the number of samples isolated from child or adult.

Supplementary file 5. Strains (global pneumococcal sequence clusters [GPSCs]) in the Maela cohort, and the number of samples isolated from child or mother.

Supplementary file 6. Serotypes and strains (global pneumococcal sequence clusters [GPSCs]) in the subset of the Maela cohort with unique samples only, and the number of samples isolated from child or mother for each, including percentages.

Supplementary file 7. Serotypes and strains (global pneumococcal sequence clusters [GPSCs]) in the subset of the Maela cohort with unique paired (mother–child) samples only, and the number of samples isolated from child or mother for each, including percentages.

Supplementary file 8. Unitigs associated with carriage age in the Dutch cohort when not corrected for population structure of the bacterial population (lrt-p-value). The other columns provide parameters of the regression line for the unitig. The final column (annotation) provides the location of the unitig in the Streptococcus_pneumoniae_D39V genome.

Supplementary file 9. Unitigs associated with carriage age in the Maela cohort when not corrected for population structure of the bacterial population (lrt-p-value). The other columns provide parameters of the regression line for the unitig. The final column (annotation) provides the location of the unitig in the Streptococcus_pneumoniae_D39V genome.

Supplementary file 10. Unitigs represent the top hits for carriage age after meta-analysis of both cohorts. These unitigs are not found in any currently available reference genome, but are found to be upstream of an accessory Sec-dependent serine-rich glycoprotein adhesin in a subset of samples from these cohorts.

Supplementary file 11. Statistics on the assembly of the sequences from the Dutch cohort.

Supplementary file 12. Sample name, sample accession, lane name, and lane accession in the European Nucleotide Archive for the sequences from the Dutch cohort.

Supplementary file 13. Sample name, sample accession, lane name, and lane accession in the European Nucleotide Archive for the sequences from the Maela cohort.

Transparent reporting form

## References

[bib1] Altschul SF, Gish W, Miller W, Myers EW, Lipman DJ (1990). Basic local alignment search tool. Journal of Molecular Biology.

[bib2] Binsker U, Lees JA, Hammond AJ, Weiser JN (2020). Immune exclusion by naturally acquired secretory IgA against pneumococcal pilus-1. The Journal of Clinical Investigation.

[bib3] Bogaert D, van Belkum A, Sluijter M, Luijendijk A, de Groot R, Rümke HC, Verbrugh HA, Hermans PWM (2004). Colonisation by Streptococcus pneumoniae and *Staphylococcus aureus* in healthy children. Lancet.

[bib4] Bosch AATM, van Houten MA, Bruin JP, Wijmenga-Monsuur AJ, Trzciński K, Bogaert D, Rots NY, Sanders EAM (2016). Nasopharyngeal carriage of Streptococcus pneumoniae and other bacteria in the 7th year after implementation of the pneumococcal conjugate vaccine in the Netherlands. Vaccine.

[bib5] Campo JJ, Le TQ, Pablo JV, Hung C, Teng AA, Tettelin H, Tate A, Hanage WP, Alderson MR, Liang X, Malley R, Lipsitch M, Croucher NJ (2018). Panproteome-wide analysis of antibody responses to whole cell pneumococcal vaccination. eLife.

[bib6] Chan JM, Gori A, Nobbs AH, Heyderman RS (2020). Streptococcal serine-rich repeat proteins in colonization and disease. Frontiers in Microbiology.

[bib7] Chewapreecha C, Harris SR, Croucher NJ, Turner C, Marttinen P, Cheng L, Pessia A, Aanensen DM, Mather AE, Page AJ, Salter SJ, Harris D, Nosten F, Goldblatt D, Corander J, Parkhill J, Turner P, Bentley SD (2014a). Dense genomic sampling identifies highways of pneumococcal recombination. Nature Genetics.

[bib8] Chewapreecha C, Marttinen P, Croucher NJ, Salter SJ, Harris SR, Mather AE, Hanage WP, Goldblatt D, Nosten FH, Turner C, Turner P, Bentley SD, Parkhill J (2014b). Comprehensive identification of single nucleotide polymorphisms associated with beta-lactam resistance within pneumococcal mosaic genes. PLOS Genetics.

[bib9] Colijn C, Corander J, Croucher NJ (2020). Designing ecologically optimized pneumococcal vaccines using population genomics. Nature Microbiology.

[bib10] Corander J, Fraser C, Gutmann MU, Arnold B, Hanage WP, Bentley SD, Lipsitch M, Croucher NJ (2017). Frequency-dependent selection in vaccine-associated pneumococcal population dynamics. Nature Ecology & Evolution.

[bib11] Cremers AJH, Mobegi FM, van der Gaast-de Jongh C, van Weert M, van Opzeeland FJ, Vehkala M, Knol MJ, Bootsma HJ, Välimäki N, Croucher NJ, Meis JF, Bentley S, van Hijum S, Corander J, Zomer AL, Ferwerda G, de Jonge MI (2019). The contribution of genetic variation of *Streptococcus pneumoniae* to the clinical manifestation of invasive pneumococcal disease. Clinical Infectious Diseases.

[bib12] Croucher NJ, Harris SR, Fraser C, Quail MA, Burton J, van der Linden M, McGee L, von Gottberg A, Song JH, Ko KS, Pichon B, Baker S, Parry CM, Lambertsen LM, Shahinas D, Pillai DR, Mitchell TJ, Dougan G, Tomasz A, Klugman KP, Parkhill J, Hanage WP, Bentley SD (2011). Rapid pneumococcal evolution in response to clinical interventions. Science.

[bib13] Croucher NJ, Finkelstein JA, Pelton SI, Mitchell PK, Lee GM, Parkhill J, Bentley SD, Hanage WP, Lipsitch M (2013). Population genomics of post-vaccine changes in pneumococcal epidemiology. Nature Genetics.

[bib14] Croucher NJ, Løchen A, Bentley SD (2018). Pneumococcal vaccines: Host interactions, population dynamics, and design principles. Annual Review of Microbiology.

[bib15] Desai AP, Sharma D, Crispell EK, Baughman W, Thomas S, Tunali A, Sherwood L, Zmitrovich A, Jerris R, Satola SW, Beall B, Moore MR, Jain S, Farley MM (2015). Decline in pneumococcal nasopharyngeal carriage of vaccine serotypes after the introduction of the 13-valent pneumococcal conjugate vaccine in children in atlanta, georgia. The Pediatric Infectious Disease Journal.

[bib16] Earle SG, Wu CH, Charlesworth J, Stoesser N, Gordon NC, Walker TM, Spencer CCA, Iqbal Z, Clifton DA, Hopkins KL, Woodford N, Smith EG, Ismail N, Llewelyn MJ, Peto TE, Crook DW, McVean G, Walker AS, Wilson DJ (2016). Identifying lineage effects when controlling for population structure improves power in bacterial association studies. Nature Microbiology.

[bib17] Ganaie F, Saad JS, McGee L, van Tonder AJ, Bentley SD, Lo SW, Gladstone RA, Turner P, Keenan JD, Breiman RF, Nahm MH (2020). A new pneumococcal capsule type, 10D, is the 100th serotype and has A large cps fragment from an oral streptococcus. MBio.

[bib18] Gladstone RA, Lo SW, Lees JA, Croucher NJ, van Tonder AJ, Corander J, Page AJ, Marttinen P, Bentley LJ, Ochoa TJ, Ho PL, du Plessis M, Cornick JE, Kwambana-Adams B, Benisty R, Nzenze SA, Madhi SA, Hawkins PA, Everett DB, Antonio M, Dagan R, Klugman KP, von Gottberg A, McGee L, Breiman RF, Bentley SD, Global Pneumococcal Sequencing Consortium (2019). International genomic definition of pneumococcal lineages, to contextualise disease, antibiotic resistance and vaccine impact. EBioMedicine.

[bib19] Gladstone RA, Lo SW, Goater R, Yeats C, Taylor B, Hadfield J, Lees JA, Croucher NJ, van Tonder AJ, Bentley LJ, Quah FX, Blaschke AJ, Pershing NL, Byington CL, Balaji V, Hryniewicz W, Sigauque B, Ravikumar KL, Almeida SCG, Ochoa TJ, Ho PL, du Plessis M, Ndlangisa KM, Cornick JE, Kwambana-Adams B, Benisty R, Nzenze SA, Madhi SA, Hawkins PA, Pollard AJ, Everett DB, Antonio M, Dagan R, Klugman KP, von Gottberg A, Metcalf BJ, Li Y, Beall BW, McGee L, Breiman RF, Aanensen DM, Bentley SD, The Global Pneumococcal Sequencing Consortium (2020). Visualizing variation within Global Pneumococcal Sequence Clusters (GPSCS) and country population snapshots to contextualize pneumococcal isolates. Microbial Genomics.

[bib20] Jaillard M, Lima L, Tournoud M, Mahé P, van Belkum A, Lacroix V, Jacob L (2018). A fast and agnostic method for bacterial genome-wide association studies: Bridging the gap between k-mers and genetic events. PLOS Genetics.

[bib21] Koelman DLH, Brouwer MC, van de Beek D (2020). Resurgence of pneumococcal meningitis in Europe and Northern America. Clinical Microbiology and Infection.

[bib22] Kremer PHC (2022a). Software Heritage.

[bib23] Kremer PHC (2022b). Software Heritage.

[bib24] Kuipers K, Lokken KL, Zangari T, Boyer MA, Shin S, Weiser JN (2018). Age-related differences in IL-1 signaling and capsule serotype affect persistence of Streptococcus pneumoniae colonization. PLOS Pathogens.

[bib25] Ladhani SN, Collins S, Djennad A, Sheppard CL, Borrow R, Fry NK, Andrews NJ, Miller E, Ramsay ME (2018). Rapid increase in non-vaccine serotypes causing invasive pneumococcal disease in England and Wales, 2000-17: a prospective national observational cohort study. The Lancet. Infectious Diseases.

[bib26] Lees JA, Vehkala M, Välimäki N, Harris SR, Chewapreecha C, Croucher NJ, Marttinen P, Davies MR, Steer AC, Tong SYC, Honkela A, Parkhill J, Bentley SD, Corander J (2016). Sequence element enrichment analysis to determine the genetic basis of bacterial phenotypes. Nature Communications.

[bib27] Lees JA, Brouwer M, van der Ende A, Parkhill J, van de Beek D, Bentley SD (2017a). Within-host sampling of a natural population shows signs of selection on pde1 during bacterial meningitis. Infection and Immunity.

[bib28] Lees JA, Croucher NJ, Goldblatt D, Nosten F, Parkhill J, Turner C, Turner P, Bentley SD (2017b). Genome-wide identification of lineage and locus specific variation associated with pneumococcal carriage duration. eLife.

[bib29] Lees JA, Kremer PHC, Manso AS, Croucher NJ, Ferwerda B, Serón MV, Oggioni MR, Parkhill J, Brouwer MC, van der Ende A, van de Beek D, Bentley SD (2017c). Large scale genomic analysis shows no evidence for pathogen adaptation between the blood and cerebrospinal fluid niches during bacterial meningitis. Microbial Genomics.

[bib30] Lees JA, Galardini M, Bentley SD, Weiser JN, Corander J (2018). pyseer: a comprehensive tool for microbial pangenome-wide association studies. Bioinformatics.

[bib31] Lees JA, Ferwerda B, Kremer PHC, Wheeler NE, Serón MV, Croucher NJ, Gladstone RA, Bootsma HJ, Rots NY, Wijmega-Monsuur AJ, Sanders EAM, Trzciński K, Wyllie AL, Zwinderman AH, van den Berg LH, van Rheenen W, Veldink JH, Harboe ZB, Lundbo LF, de Groot L, van Schoor NM, van der Velde N, Ängquist LH, Sørensen TIA, Nohr EA, Mentzer AJ, Mills TC, Knight JC, du Plessis M, Nzenze S, Weiser JN, Parkhill J, Madhi S, Benfield T, von Gottberg A, van der Ende A, Brouwer MC, Barrett JC, Bentley SD, van de Beek D (2019a). Joint sequencing of human and pathogen genomes reveals the genetics of pneumococcal meningitis. Nature Communications.

[bib32] Lees JA, Harris SR, Tonkin-Hill G, Gladstone RA, Lo SW, Weiser JN, Corander J, Bentley SD, Croucher NJ (2019b). Fast and flexible bacterial genomic epidemiology with PopPUNK. Genome Research.

[bib33] Lees JA, Mai TT, Galardini M, Wheeler NE, Horsfield ST, Parkhill J, Corander J (2020). Improved prediction of bacterial genotype-phenotype associations using interpretable pangenome-spanning regressions. MBio.

[bib34] Li Y, Metcalf BJ, Chochua S, Li Z, Walker H, Tran T, Hawkins PA, Gierke R, Pilishvili T, McGee L, Beall BW (2019). Genome-wide association analyses of invasive pneumococcal isolates identify a missense bacterial mutation associated with meningitis. Nature Communications.

[bib35] Lippert C, Casale FP, Rakitsch B, Stegle O (2014). LIMIX: Genetic Analysis of Multiple Traits. bioRxiv.

[bib36] Malley R, Lipsitch M, Stack A, Saladino R, Fleisher G, Pelton S, Thompson C, Briles D, Anderson P (2001). Intranasal immunization with killed unencapsulated whole cells prevents colonization and invasive disease by capsulated pneumococci. Infection and Immunity.

[bib37] Middleton DR, Aceil J, Mustafa S, Paschall AV, Avci FY (2021). Glycosyltransferases within the *psrP* locus facilitate pneumococcal virulence. Journal of Bacteriology.

[bib38] Moffitt K, Malley R (2016). Rationale and prospects for novel pneumococcal vaccines. Human Vaccines & Immunotherapeutics.

[bib39] Morais V, Texeira E, Suarez N (2019). Next-Generation Whole-Cell Pneumococcal Vaccine. Vaccines.

[bib40] Nelson AL, Roche AM, Gould JM, Chim K, Ratner AJ, Weiser JN (2007). Capsule enhances pneumococcal colonization by limiting mucus-mediated clearance. Infection and Immunity.

[bib41] Nguyen L-T, Schmidt HA, von Haeseler A, Minh BQ (2015). IQ-TREE: A fast and effective stochastic algorithm for estimating maximum-likelihood phylogenies. Molecular Biology and Evolution.

[bib42] O Brien KL, Nohynek H, Watt JP, World Health Organization Pneumococcal Vaccine Trials Carriage Working Group (2003). Report from a WHO Working Group: standard method for detecting upper respiratory carriage of Streptococcus pneumoniae. The Pediatric Infectious Disease Journal.

[bib43] O Brien KL, Wolfson LJ, Watt JP, Henkle E, Deloria-Knoll M, McCall N, Lee E, Mulholland K, Levine OS, Cherian T, Hib and Pneumococcal Global Burden of Disease Study Team (2009). Burden of disease caused by streptococcus pneumoniae in children younger than 5 years: global estimates. Lancet.

[bib44] Page AJ, Cummins CA, Hunt M, Wong VK, Reuter S, Holden MTG, Fookes M, Falush D, Keane JA, Parkhill J (2015). Roary: rapid large-scale prokaryote pan genome analysis. Bioinformatics.

[bib45] Page AJ, De Silva N, Hunt M, Quail MA, Parkhill J, Harris SR, Otto TD, Keane JA (2016). Robust high-throughput prokaryote *de novo* assembly and improvement pipeline for Illumina data. Microbial Genomics.

[bib46] Pichichero ME (2017). Pneumococcal whole-cell and protein-based vaccines: changing the paradigm. Expert Review of Vaccines.

[bib47] Poehling KA, Talbot TR, Griffin MR, Craig AS, Whitney CG, Zell E, Lexau CA, Thomas AR, Harrison LH, Reingold AL, Hadler JL, Farley MM, Anderson BJ, Schaffner W (2006). Invasive pneumococcal disease among infants before and after introduction of pneumococcal conjugate vaccine. JAMA.

[bib48] Schweiger R, Fisher E, Rahmani E, Shenhav L, Rosset S, Halperin E (2018). Using stochastic approximation techniques to efficiently construct confidence intervals for heritability. Journal of Computational Biology.

[bib49] Seemann T (2014). Prokka: rapid prokaryotic genome annotation. Bioinformatics.

[bib50] Spijkerman J, Prevaes S, van Gils EJM, Veenhoven RH, Bruin JP, Bogaert D (2012). Long-term effects of pneumococcal conjugate vaccine on nasopharyngeal carriage of S pneumoniae, S aureus, H influenzae and M catarrhalis. PLOS ONE.

[bib51] Stearns JC, Davidson CJ, McKeon S, Whelan FJ, Fontes ME, Schryvers AB, Bowdish DME, Kellner JD, Surette MG (2015). Culture and molecular-based profiles show shifts in bacterial communities of the upper respiratory tract that occur with age. The ISME Journal.

[bib52] Trzciński K, Bogaert D, Wyllie A, Chu MLJN, van der Ende A, Bruin JP, van den Dobbelsteen G, Veenhoven RH, Sanders EAM (2013). Superiority of trans-oral over trans-nasal sampling in detecting Streptococcus pneumoniae colonization in adults. PLOS ONE.

[bib53] Turner P, Melchiorre S, Moschioni M, Barocchi MA, Turner C, Watthanaworawit W, Kaewcharernnet N, Nosten F, Goldblatt D (2012a). Assessment of Streptococcus pneumoniae pilus islet-1 prevalence in carried and transmitted isolates from mother-infant pairs on the Thailand-Burma border. Clinical Microbiology and Infection.

[bib54] Turner P, Turner C, Jankhot A, Helen N, Lee SJ, Day NP, White NJ, Nosten F, Goldblatt D (2012b). A longitudinal study of Streptococcus pneumoniae carriage in A cohort of infants and their mothers on the Thailand-Myanmar border. PLOS ONE.

[bib55] Turner P, Turner C, Jankhot A, Phakaudom K, Nosten F, Goldblatt D (2013). Field evaluation of culture plus latex sweep serotyping for detection of multiple pneumococcal serotype colonisation in infants and young children. PLOS ONE.

[bib56] van Beek J, Veenhoven RH, Bruin JP, van Boxtel RAJ, de Lange MMA, Meijer A, Sanders EAM, Rots NY, Luytjes W (2017). Influenza-like illness incidence is not reduced by influenza vaccination in a cohort of older adults, despite effectively reducing laboratory-confirmed influenza virus infections. The Journal of Infectious Diseases.

[bib57] von Gottberg A, de Gouveia L, Tempia S, Quan V, Meiring S, von Mollendorf C, Madhi SA, Zell ER, Verani JR, O’Brien KL, Whitney CG, Klugman KP, Cohen C, GERMS-SA Investigators (2014). Effects of vaccination on invasive pneumococcal disease in South Africa. The New England Journal of Medicine.

[bib58] Wahl B, O’Brien KL, Greenbaum A, Majumder A, Liu L, Chu Y, Lukšić I, Nair H, McAllister DA, Campbell H, Rudan I, Black R, Knoll MD (2018). Burden of Streptococcus pneumoniae and Haemophilus influenzae type b disease in children in the era of conjugate vaccines: global, regional, and national estimates for 2000-15. The Lancet. Global Health.

[bib59] Weiser JN, Ferreira DM, Paton JC (2018). Streptococcus pneumoniae: transmission, colonization and invasion. Nature Reviews. Microbiology.

[bib60] Whitney CG, Farley MM, Hadler J, Harrison LH, Bennett NM, Lynfield R, Reingold A, Cieslak PR, Pilishvili T, Jackson D, Facklam RR, Jorgensen JH, Schuchat A, Active Bacterial Core Surveillance of the Emerging Infections Program Network (2003). Decline in invasive pneumococcal disease after the introduction of protein-polysaccharide conjugate vaccine. The New England Journal of Medicine.

[bib61] Willer CJ, Li Y, Abecasis GR (2010). METAL: fast and efficient meta-analysis of genomewide association scans. Bioinformatics.

[bib62] Wyllie AL, Wijmenga-Monsuur AJ, van Houten MA, Bosch AATM, Groot JA, van Engelsdorp Gastelaars J, Bruin JP, Bogaert D, Rots NY, Sanders EAM, Trzciński K (2016). Molecular surveillance of nasopharyngeal carriage of Streptococcus pneumoniae in children vaccinated with conjugated polysaccharide pneumococcal vaccines. Scientific Reports.

[bib63] Wyllie AL, Warren JL, Regev-Yochay G, Givon-Lavi N, Dagan R, Weinberger DM (2020). Serotype patterns of pneumococcal disease in adults are correlated with carriage patterns in older children. Clinical Infectious Diseases: An Official Publication of the Infectious Diseases Society of America.

[bib64] Young BC, Golubchik T, Batty EM, Fung R, Larner-Svensson H, Votintseva AA, Miller RR, Godwin H, Knox K, Everitt RG, Iqbal Z, Rimmer AJ, Cule M, Ip CLC, Didelot X, Harding RM, Donnelly P, Peto TE, Crook DW, Bowden R, Wilson DJ (2012). Evolutionary dynamics of *Staphylococcus aureus* during progression from carriage to disease. PNAS.

[bib65] Zafar MA, Hammond AJ, Hamaguchi S, Wu W, Kono M, Zhao L, Weiser JN (2019). Identification of pneumococcal factors affecting pneumococcal shedding shows that the *dlt* locus promotes inflammation and transmission. MBio.

